# Bioinspired Dry Adhesives for Highly Adaptable and Stable Manipulating Irregular Objects under Vibration

**DOI:** 10.1002/advs.202302512

**Published:** 2023-05-07

**Authors:** Duorui Wang, Hongmiao Tian, Haoran Liu, Jinyu Zhang, Hong Hu, Xiangming Li, Chunhui Wang, Xiaoliang Chen, Jinyou Shao

**Affiliations:** ^1^ Micro‐and Nano‐Technology Research Center State Key Laboratory for Manufacturing Systems Engineering Xi'an Jiaotong University Xi'an Shaanxi 710049 China; ^2^ Frontier Institute of Science and Technology (FIST) Xi'an Jiaotong University Xi'an Shaanxi 710049 China; ^3^ Institute of Textiles and Clothing The Hong Kong Polytechnic University Hong Kong SAR 100872 China

**Keywords:** bioinspired adhesive materials, contact adaptability, irregular surfaces, multi‐scale bionics, stable adhesion

## Abstract

For dry adhesive–based operation, highly adaptable and stable manipulation is important but also challenging, especially for irregular objects with complex surface (such as abrupt profile and acute projection) under vibration‐inducing environments. Here, a multi‐scale adhesive structure, with mechanically isolated energy‐absorbing backing, based on the synergistic action of microscale contact end (seta), mesoscale supporting layer (lamella), and macroscopic backing (muscle tissues) of gecko's sole, is proposed. Top layer of mushroom‐like micro tips provides dry adhesion via mimicking gecko's seta, and bottom layer of physical cuts and porous feature achieves the interfacial mechanical decoupling and crack inhibition via mimicking the non‐continuous distributing of lamella and compliance of muscle. The proposed dry adhesive exhibits excellent adaptable adhesion to various objects with curved or irregular surfaces, even for that with abrupt contours, as well as an amazing stable anti‐vibration ability, opening a new avenue for the development of dry adhesive–based device or system.

## Graphical abstract

Inspired by the synergistic influence of microscale contact end (seta), mesoscopic supporting layer (lamella), and macro backing (muscle tissues) of gecko's sole, a multi‐scale bionic dry adhesive is proposed. It is experimentally demonstrated to have adaptable adhesion to various objects with curved or irregular surfaces, even for that with abrupt contours, as well as an amazing stable anti‐vibration ability.

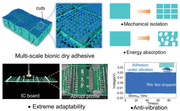

## 1 Introduction

Dry adhesive materials exhibit great potential in the fields of transport manipulators,^[^ ^1^, ^2^, ^3^, ^4^, ^5^, ^6^, ^7^, ^8^, ^9^ ^]^ climbing robots,^[^ ^10^, ^11^, ^12^, ^13^ ^]^ biological patches,^[^ ^14^, ^15^, ^16^, ^17^, ^18^ ^]^ etc., due to the interfacial action on the adhered interfaces, that are mainly depended on van der Waals force with the superior adaptability on materials, texture, etc.^[^ ^19^, ^20^, ^21^ ^]^ Despite significant progress has been achieved from the viewpoints of structural optimization and materials exploration, the adaptability and stability of dry adhesives still confront great challenges. For instance, regarding the objects with complex surfaces (such as abrupt profile and acute projection), the conformal contact is difficult to be obtained due to the mismatch between the adhesive structure and the opposing surface. The stress concentration caused by poor contact would easily enhance crack propagation and weaken the adhesion. In addition, vibration is commonly performed in the operation process of robotic system, maybe leading to the puzzle of dynamic stability. Even for a slight vibration, the interfacial crack may occur rapidly, spread to the whole adhered interface and destroy the adhesion. Inhibitory action of vibration on adhesion is necessary for practical adhesive behavior, which, however, is seldom explored in the published works according to our best knowledge. Consequently, improving the adaptability to irregular surfaces while maintaining stable anti‐vibration ability is critical to promote the application horizon of artificial dry adhesives.

Various strategies have been developed for enhancing surface adaptability via hierarchical design, composite structure, or deformation control. For hierarchical structure,^[^ ^22^, ^23^, ^24^, ^25^, ^26^, ^27^, ^28^, ^29^, ^30^ ^]^ the key consideration is optimizing structural geometry and reducing the effective elastic modulus to improve the conformal contact between adhesive structure and opposing surface. Regarding composite structure with a stiffness gradient (such as soft shell and rigid core), the interfacial crack propagation can be suppressed via adjusting interface stress distribution.^[^ ^31^, ^32^, ^33^, ^34^, ^35^ ^]^ These approaches actually increase the adhesive performance on rough surface, which, however, are mainly effective on roughness of microscale and still demonstrates poor adhesion on the surfaces with extreme morphology (step profile, acute projection, for instance). Additionally, the conception of deformation control, such as pneumatically‐based dry adhesive, can markedly improve the adaptability to large‐scale 3D surfaces by stiffness regulation,^[^ ^3^, ^8^ ^]^ which appear inability on the object with mutant characteristics due to the localized stress concentrations in adhered interface. As for the adhesion stability, there seems scarcely any exploration on the influence of vibration on adhesive performance, which however is a critical factor on dry adhesive–based operation. Notably, the aforementioned explorations are mainly performed on the basis of inspiration on the terminal structure of gecko's toe.

The optimal design of terminal micro‐structure alone seems impossible to satisfy the requirements of adaption on complex irregular surfaces and adhesion stability, that is, the action incurred by the elastic communication of the fibrils through the backing layer cannot be ignored. Especially, even if the conformal contact can be obtained by adjusting the stiffness of micro‐end structures, an emergent adhesion damage (a small interfacial crack, for instance) at a certain position would be quickly transmitted through the backing layer to the whole adhered interface, maybe leading to the failure of dry adhesion, which acts similar to that of peeling‐off behavior. Under the consideration of spread behavior of interfacial crack, the contact splitting effect (i.e., fibrillary‐arrays) can decouple the interaction between different fibers.^[^ ^36^ ^]^ However, recent adhesive structures proposed from the contact splitting effect are also mostly concentrated on the micro‐end structures, which is convenient for improving the adhesion on irregular surfaces with roughness at nano/microscale but impossible for large‐scale uneven surfaces (hundreds of micrometers or even larger), let alone mutant surfaces. More importantly, structural design only on the micro‐end layer cannot promote the performance of adhesion stability. Although a great deal of work has been done to improve adaptability by introducing soft foam backing into adhesion‐based robotic systems,^[^ ^11^, ^37^, ^38^, ^39^ ^]^ it is still difficult to avoid the problem of cracks spreading easily due to continuous interfaces. In this case, even if an ideal contact is established based on the compliance of the soft backing, the low tolerance of the continuous adhesion interface to defects results instable gripping of target objects with complex abrupt contours.

**Figure 1:**
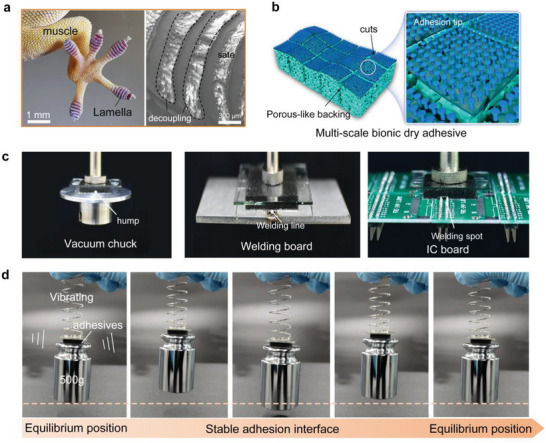
Design of MIEA‐DA. a) Structural morphology of the soles of gecko's sole; b) the structural description of the MIEA‐DA; c) the adaptability of MIEA‐DA to different objects with abrupt contours; and d) performance of MIEA‐DA under a simulate vibration environment.

In contrast, gecko can climb on various surfaces with the roughness from nanoscale to microscale or even to macroscale, and could stably attach on moving or vibrant objects (such as swinging boughs and bumped cars), demonstrating excellent surface adaptability and attachment stability. Careful observation of the gecko's soles shows that, in addition to the highly dispersed microscale setal arrays, several rows of mesoscale lamella are also isolated (**Figure** **1**a). This feature can effectively realize the interfacial mechanical decoupling on the macro scale, which can further improve the adaptability to complex non‐smooth surfaces based on ideal contact contributed by the setal arrays. In addition, the high‐elastic underlying muscle tissue, similar to a soft buffer, can absorb the harmful shock from the external environment during the crawling process and ensure the stability of interfacial adhesion. Hence, a multi‐scale dry adhesive with high adaptability and stability is proposed based on mechanically isolated energy‐absorbing backing, that is, mechanically isolated energy‐absorbing dry adhesives (MIEA‐DA), as shown in Figure 1b. The end micro contacts adopt a mushroom‐shaped structure to provide strong adhesion like seta.^[^ ^40^, ^41^, ^42^, ^43^ ^]^ Physical cuts were introduced to discrete the continuous surface layer into several adhesion units like lamella, realizing the mechanical decoupling of each independent unit. Here, the connected backing layer was designed as a porous network to mimic the muscle structure, thereby effectively reducing the contact stiffness.

## 2 Results and Discussion

### 2.1 Demonstration of Multi‐Level Bionic Adhesive

The fabrication process of MIEA‐DA was mainly based on the molding process, as detailed in Figure S1, Supporting Information. The mold with mushroom‐like cavity was prepared by the double‐sided exposure process proposed by our team before,^[^ ^44^ ^]^ which can realize the fabrication of mushroom‐shaped structure with controllable topography and good uniformity. The porous backing adopts polyurethane foam with dimensions of 2 cm × 2 cm × 4 mm and an elastic modulus of ≈80 kPa (Figure S2, Supporting Information). The pore size inside the porous backing is about 500 µm and the diameter of the fiber skeleton inside porous structure is about 100 µm. It can be clearly seen from the scanning electron microscope (SEM) images that MIEA‐DA is composed of three parts, that is, the mushroom‐shaped microstructure on the top layer, a thin adhesive film separated by cuts, and a porous backing layer (Figure S3, Supporting Information). The mushroom‐shaped tip was distributed in a square shape (Figure S4, Supporting Information), and the diameter, height and spacing were 18 µm, 14 µm and 20 µm, respectively.

The coupling effect of physical cuts and porous network characteristics endow the MIEA‐DA with two important characteristics. First, the existence of cuts and pores enhances the compressibility of adhesives. Therefore, the adhesives can be well adapted to the surface with complex contours and shapes (Figure S5, Supporting Information). Although traditional soft adhesives can still rely on their low modulus and compliance to achieve adaptive contact with the target profile, the continuous soft adhesion interface has poor inhibition of crack propagation, resulting in their inability to complete stable pickup of the target (Movie S1, Supporting Information). Unlike traditional soft dry adhesives, the cuts eliminate the possibility of stress concentration at the transition of mutant profile by mechanically isolating several adhesion units. This ability enables the proposed adhesive to stably pick up objects with abrupt profiles, such as vacuum chuck, welding pads, and IC circuit boards, which are quite common in the industry (Figure 1c). Here, the gripping of the pure porous backing–based adhesives for these complex objects was also verified (Movie S2, Supporting Information). Since the continuous adhesion interface is difficult to effectively resist the peeling caused by local stress concentration, the pure porous backing‐based adhesives cannot achieve stable gripping for IC circuit board and vacuum chuck with more obvious mutant profile (Figure S6, Supporting Information), which further proves that the proposed multi‐scale bioinspired adhesives with interface mechanical decoupling characteristics is fundamentally different from the traditional soft soam–based adhesives. Second, the pores can act as a buffer to absorb the energy generated by external vibrations. Even if a part of the contact interface was separated during vibration process, the existence of cuts can minimize the impact of non‐contact area on the contact area and ensure the stability of the adhesion system. That is, the energy absorption characteristics given by the porous feature and the crack inhibition effect given by the mechanical isolation have dual effects on the anti‐vibration ability. A simple vibration system by the spring was built to verify the anti‐vibration performance of the adhesive (Movie S3, Supporting Information). With reciprocating vibrations of the target object near the equilibrium position, the adhesion state is always stable and reliable, reflecting the high anti‐vibration ability of MIEA‐DA (Figure 1d).

### 2.2 Adhesion Performance

**Figure 2:**
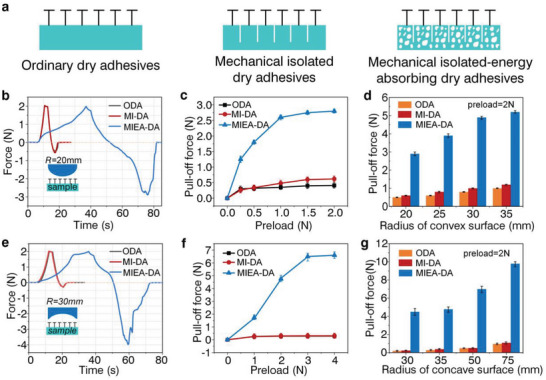
The normal adhesion strength of different adhesives to convex and concave surfaces. a) The structural form of ODA, MI‐DA, and MIEA‐DA; b) the load–pull curves of a cylindrical probe with a radius of 20 mm to three structures; c) the normal adhesion of three structures to the cylindrical probe (*R* = 20 mm) under different preloads; d) the normal adhesion strength of different structures to cylindrical probes with different radii; e) The load–pull curves of a concave probe with a radius of 30 mm to three structures; f) the normal adhesion of three structures to concave probe (*R* = 30 mm) under different preloads; and g) the normal adhesion strength of different structures to concave probes with different radii.

In order to verify the contact adaptability of MIEA‐DA, typical convex and concave surfaces (Figure S7, Supporting Information) were employed to assess the normal adhesion strength of the adhesive. Herein, ordinary dry adhesives (ODA) and mechanically isolated dry adhesives (MI‐DA) were fabricated to assess the performance difference (**Figure** **2**a). The testing was carried out in a pull‐pressure testing machine, and the test speed during the preload and pull process was 5 mm min^−1^ (Figure S8, Supporting Information). Figure 2b shows the load–pull curves of a convex probe with a radius of 20 mm to three adhesives. It can be seen that MIEA‐DA shows better adhesion performance than ODA and MI‐DA under the same preload (2 N). Besides, relying on the cuts alone cannot significantly improve the adhesion performance, but requires further structural design of the connected backing layer. In addition, the curve of MIEA‐DA during the pulling stage exhibits a zigzag shape, which also indicates that the interfacial separation process is not continuous due to mechanically isolated units. Figure 2c shows the normal adhesion strength of three structures to the convex probe under different preloads, which also confirms the excellent performance of MIEA‐DA. It is noteworthy that the strategy of employing mechanically isolated energy‐absorbing backing not only improves the adhesion strength, but also increases the ratio of adhesive force to preload (up to 6), while the other two structures are less than 1. In other words, MIEA‐DA can achieve a large adhesive strength under a small preload, which is important in some load‐sensitive applications. This advantage is not only due to the lower elastic modulus, but also related to the unique mechanical properties of porous materials under compression. The stress–strain curve of porous materials undergoes a plateau region under compression, which is manifested by the load maintaining a stable value with the increase of deformation (Figure S2, Supporting Information). Convex probes with different radii are used to test the normal adhesion strength of different structures (Figure 2d). Compared with ODA, the adhesion performance of MIEA‐DA can be improved by five times.

Moreover, concave surfaces, another type of uneven surface, are commonly used, which are difficult for conformal contact. Figure 2e describes the load–pull curves of three structures on a concave surface with a radius of 30 mm. Like the convex surface, MIEA‐DA also shows excellent performance compared to the other two structures. It is worth noting that the adhesion of ODA and MI‐DA is always lower than the convex surface under the same preload, which is due to the different contact modes of characteristic surfaces (Figure S9, Supporting Information). During the contact process of the convex surface, the adhesive structure starts to fit from the middle region to both sides. In contrast, the adhesive structure starts to fit from both sides to the middle during the contact process of concave surface, which undoubtedly increases the contact difficulty. Therefore, when the specified pre‐pressure is reached, the ODA and MI‐DA only contact both sides of the area on the inner concave, where stress concentration is inevitably greater than the convex surface, leading to the rapid peeling and decreasing the adhesive force. On the other hand, the interfaces of both concave and convex surfaces are completely fitted to MIEA‐DA because the modulus is reduced due to the coupling of cuts and pores. Therefore, when the radius of concave surface is larger than the convex surface, the adhesion of MIEA‐DA to the concave surface is higher than the convex surface.

In the same way, normal adhesion under different preload conditions is analyzed, as shown in Figure 2f. ODA and MI‐DA are maintained at a low level, while the adhesive force of MIEA‐DA gradually increases with the increase of preload, reaching maximum value under a preload of 4 N. Of course, the ratio of adhesion to preload of MIEA‐DA is lower than the convex surface due to the difference in contact modes, but it is still much higher than ODA and MI‐DA. Figure 2g presents the adhesion of three structures on concave surfaces with different radii. Compared with the ODA, the performance of MIEA‐DA is improved by about ten times. Here, the adhesion performance of purely porous backing–based adhesives was also compared with that of MIEA‐DA on non‐flat surfaces (Figure S10, Supporting Information), and the results showed that the adhesive force of uncut porous backing to concave and convex surfaces with different curvatures are worse than that of MIEA‐DA, which further demonstrates the importance of mechanical isolation.

### 2.3 Adhesion Enhancement Mechanism

**Figure 3:**
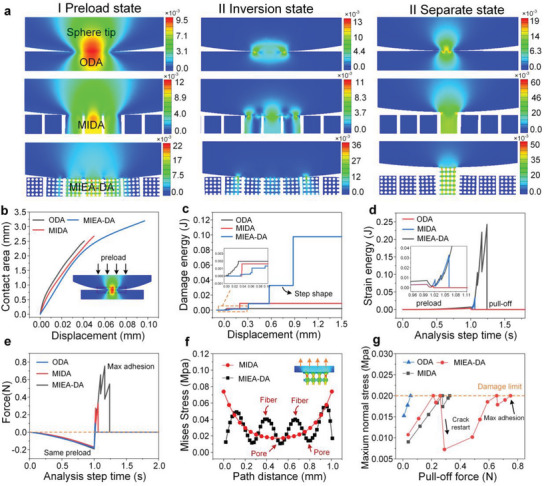
The simulation results of adhesion on a curved surface. a) The numerical simulation results of the whole process of normal adhesion of three structures to a spherical surface; b) the contact area of three structures to a spherical surface during the loading stage; c) the changes in the damage energy of three structures during the whole process; d) the changes in strain energy with respect to time during the whole process; e) the adhesion force via step time of the three structures; f) the interfacial stress distribution of DA and MIEA‐DA at the maximum adhesion moment; and g) the maximum normal stress of three structures with the pull‐off force.

To further understand the adhesion enhancement due to porous morphology and physical cuts, a numerical model based on interfacial cohesive zone theory is employed to analyze the contact–separation process of three different structures. Considering the biomimetic adhesive materials inspired by reptiles are often based on the action mechanism of van der Waals forces, a zero‐thickness cohesive surface was used for finite element simulation in this paper. The geometric model was a 2D form, which mainly includes sphere probe and backing layer. In the finite element simulation, the same preload application can be achieved by adjusting the displacement of the loading stage to simulate the test situation in the experiment. In this model, three stages were adopted to evaluate the adhesive status: stage I—preload state (moment of the maximum deformation to demonstrate the contact state); stage II—inversion state (moment of the reversal interfacial stress to demonstrate the working region on adhesion); stage III—separate state (moment of the adhesive material separated from the probe to demonstrate the output adhesive force) (**Figure** **3**a). Furthermore, the numerically simulated dynamic behavior is shown in Movies S4–S6, Supporting Information.

The contact area of MIEA‐DA is larger than the OA and DA with an identical preload due to the low elastic modulus, as shown in Figure 3a (stage I). Also, the evolution of contact area is demonstrated in Figure 3b. In this stage, elastic modulus plays a critical role and the coupling effect of physical cuts and pores undoubtedly results in an optimal contact state of MIEA‐DA. During the unloading stage, the elastic energy stored in three structures is gradually released with the recovery of deformation and the structures start to exhibit a tensile behavior, as shown in Figure 3a (stage II). The contact area of OA at the inversion stage is obviously lower than the loading stage, which means that the peeling occurs prematurely and partially compromises the contact formed during the loading stage, which can also be quantitatively described by the damage energy (Figure 3c). It can be seen that the interface of OA first produces the damage and rapidly reaches a stable peak value (complete separation), while the damage process of DA and MIEA‐DA is relatively slow, especially the latter does not completely separate until the tensile displacement of 0.9 mm.

Moreover, the damage curves of DA and MIEA‐DA exhibit obvious step‐like characteristics, that is, the fracture energy tends to be stable at a certain point, increases rapidly and, then, tends to be stable again. This means that the crack growth is a discontinuous process, which is consistent with the testing curve of adhesive force in Figure 2. This discontinuous behavior is also observed during the change in strain energy (Figure 3d), which is similar to the crack trapping effect generated by pores during interfacial adhesion, as observed by Glassmaker et al.^[^ ^29^ ^]^ Therefore, when the maximum adhesion force is reached (stage III), both DA and MIEA‐DA form a stable tensile state of a single discrete unit, which is favorable for adhesion enhancement. However, the crack trapping effect of MIEA‐DA is not only derived from pores, but is also related to the physical cuts. Specifically, when the crack expands to the cuts, the stored strain energy is not enough to provide the surface energy required for interfacial separation in front of a crack. Hence, an additional external force is required to perform the required work, which ultimately increases the adhesion force. In addition, the improvement of adaptability caused by porous characteristics also increases the contact area under the same preload compared with the ordinary substrate, which leads to the suppression and restart of cracks by more physical cuts in the separation process, that is, the crack trapping effect is further enhanced. Consequently, the adhesion force of MIEA‐DA is significantly improved compared with OA and DA, which is consistent with experimental results (Figure 3e).

In addition to the crack trapping mechanism, the existence of pores can further optimize the interfacial stress distribution. The interfacial stress distribution of MIEA‐DA and DA at the state of maximum adhesion is shown in Figure 3f. There is an obvious stress concentration on both sides of DA's contact area due to the influence of spherical probe curvature, which leads to rapid crack propagation. However, this feature is greatly weakened on the interface of MIEA‐DA. The existence of pores makes the stress distribution of the whole interface sinusoidal, which reduces the stress concentration and flattens the stress, which is undoubtedly another factor for the adhesion enhancement.

Furthermore, as the structure is stretched, each element at the interface is subjected to a load dominated by the normal force (Figure S11, Supporting Information), and the maximum value changes continuously until the damage limit (parameter is set as 0.02) is reached. The maximum normal stress of three structures with the load variation is plotted, as shown in Figure 3g. The crack propagation of OA is extremely rapid, so the maximum normal stress is soon reached the damage limit and the maximum adhesive force is also generated (*x*‐axis: 0.06 N). The initial maximum normal stress of MIEA‐DA is larger than DA and the crack trapping effect occurs after reaching the damage limit. The crack propagation was inhibited and restarted, causing the maximum normal stress decreased and then increased again. At this moment, it can be seen that the maximum normal stress of MIEA‐DA decreases more significantly due to the synergistic influence of physical cuts and pores, and a longer time is required to reach the damage parameter, resulting in a greater adhesion (0.75 N). In addition, the network‐like structure of porous materials leads to anisotropic mechanical properties, demonstrating that the tensile modulus is higher than the compressive modulus (Figure S12, Supporting Information). This feature is undoubtedly conducive to balancing a soft fit during contact and anti‐peeling effect during tension, which also renders a positive effect on adhesion.

### 2.4 Compensation Effect of MIEA‐DA on Contact Interface Error

**Figure 4:**
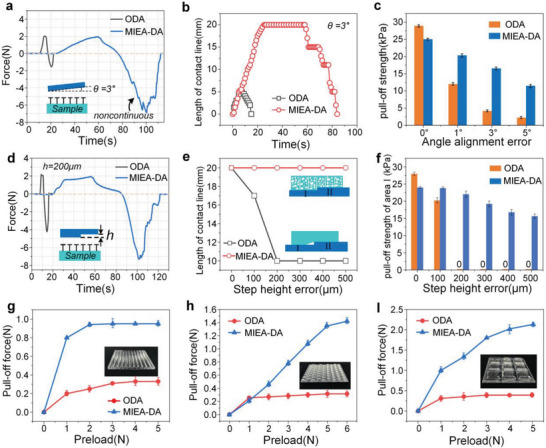
The adaptability of adhesive structure to interface error. a) The force versus time curve during the loading–pulling process when there is an angular error (3°) between the target surface and adhesion structure; b) the change in contact length with time using CCD observations; c) the adaptability of both structures to different angle errors; d) the loading–pulling curves of the adhesive structures on a step surface (200 µm); e) the contact length of both structures on step surfaces with different heights; f) the adhesion strength to the stepped surfaces with different heights; and g–i) the adhesion strength to an array‐structured PMMA.

In addition to the adhesion enhancement to the smooth curved surface, the effective adaptation to the target surface with abrupt profile is more challenging. In order to verify the adaptability of MIEA‐DA to the abrupt surfaces, the interfacial angle error and vertical error were artificially set here, and the adhesion performance of MIEA‐DA to these complex surfaces was further evaluated by characterizing the interfacial contact behavior and pull‐off force. **Figure** **4**a shows the force–time curve during the loading–pulling process when there is an angle error (3°) between the target surface and adhesive structure, and the entire interfacial separation process was recorded by CCD (Figure S13 and Movie S7, Supporting Information). The presence of both cuts and pores endows the MIEA‐DA with good adaptability to angle errors and a non‐continuous crack propagating feature is also observed during the pulling stage due to the presence of physical cuts. In order to explain more intuitively the adhesion process of MIEA‐DA, the changes in the contact length with time are observed, as shown in Figure 4b. It can be seen that the contact line of both structures increases linearly with time during the loading phase. When a threshold loading force is reached, the contact length of ODA does not reach the conformal contact state, that is, the sample width. In the case of MIEA‐DA, the loading phase takes the longest time and the final contact length reaches 20 mm, indicating a perfect fit with the target. During the unloading phase, the length of ODA contact line rapidly decreases with time, indicating that the separation process is rapid and continuous. On the contrary, an obvious step feature appears in the contact length curve of MIEA‐DA, which indicates that the physical cuts suppress the crack propagation. Figure 4c shows the adaptability of both structures to different angle errors. Overall, MIEA‐DA demonstrates excellent performance, which can be increased by up to five times compared to ODA.

In addition to the angle error, the vertical error also affects the actual adhesion effect of the adhesive materials. Figure 4d presents the loading–pulling curves of the ODA and MIEA‐DA on a step surface (*h* = 200 µm), and the entire interfacial separation process was recorded by CCD (Movie S8, Supporting Information). During the test, the transition line of the step is aligned with the centerline of the adhesive structure. Similar to the angle error, MIEA‐DA also shows the optimal performance, which is about two times higher than the ODA. It is worth noting that the contact area during the loading process is the key to the adhesion effect when there is a vertical error in the contact interface. Also, the discontinuous separation process due to the presence of cuts plays a relatively weak role here. Here, the contact state of the adhesives between step surfaces with different heights at the same preload is recorded by CCD (Figure S14, Supporting Information). When a step height of 100 µm, the contact length of ODA on a step surface is lower than the flat surface, whereas the contact length of MIEA‐DA remains constant on both types of surfaces (Figure 4e). As the height of steps gradually increases, the contact length of ODA decreases rapidly until it is maintained at 10 mm, which means that ODA cannot adapt to the steps.

On the contrary, the contact length of MIEA‐DA remains at 20 mm due to the independent contact states of discrete adhesion units. Based on the contact length, we can assess the adhesion strength of both structures to the step surface. In order to clearly illustrate the adhesion ability, the contact area used in the calculation is Region‐I and the value of adhesion force is the maximum value of region I before it is completely separated, as shown in Figure 4f. It can be seen that, except that the adhesion strength of ODA under a flat surface is slightly higher than MIEA‐DA, the adhesion strength of MIEA‐DA is much higher than ODA in the presence of steps. When the step height is 200–500 µm, the adhesion strength of ODA becomes zero due to inadaptability. However, the adhesion strength of MIEA‐DA is maintained at higher values due to the conformal contact to the step surface. The slight decrease is due to the excessive compression of the higher surface affecting the contact state. In order to verify the adhesion performance of MIEA‐DA to several uneven surfaces, the adhesion strength to PMMA with the characteristics of an array structure is tested, as shown in Figure 4g–i. The structural characterization of PMMA plate can be seen in Figure S15, Supporting Information. These results prove that MIEA‐DA renders excellent performance, which is up to seven times higher than ODA.

### 2.5 Structure Optimization

**Figure 5:**
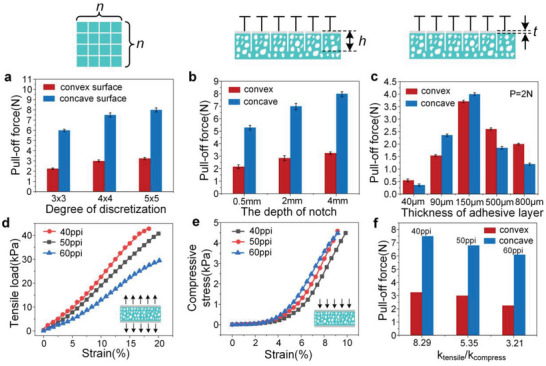
Influence of structural characteristics on adhesion performance. a) The effect of 2D discretization of the surface layer on adhesion strength; b) the adhesion strength on an uneven surface; c) the influence of adhesive layer thickness on adhesion performance; d,e) the tensile and compressive elastic moduli of porous structures with different porosities; and f) the adhesion strength of structures with different porosities to uneven surfaces.

In order to further optimize the structure to achieve better performance, it is necessary to explore the influence of process parameters and structural characteristics on adhesion strength. **Figure** **5**a presents the effect of degree of discretization (*n* × *n*) on adhesion strength. Three samples with different discrete units were tested on typical non‐flat surfaces under the same preload (2 N). Obviously, the higher degree of discretization indicates the presence of more independent discrete units, resulting in better interface adaptability. This is similar to the scaling effect mentioned by Arzt et al., which considers that the total adhesion of adhesive material is proportional to the number of splits.^[^ ^45^ ^]^ In addition to the degree of discretization, the depth of cuts (*h*) is also a key parameter, which determines the effect of mechanical isolating between different units. As shown in Figure 5b, the sample with a cut depth of 4 mm shows the most excellent adhesion performance. Owing to the complete cutting (backing layer thickness: 4 mm), each unit is completely independent and does not affect others during pull‐up, resulting in excellent adaptability and discontinues crack propagation.

Figure 5c presents the influence of top layer thickness (*t*) on the adhesion performance, which is achieved by changing the spin‐coating parameters. Interestingly, the relationship between thickness and adhesion is not simply linear but similar to the normal distribution, which can be attributed to the trade‐off between the mechanical properties of top layer and bonding state between interfaces. Specifically, the excessive thickness of top layer increases the bending stiffness and reduces the adaptability of the interface. However, the bonding strength between film and inner fiber is compromised when thickness is further reduced, thereby weakening the adhesive strength. Since the diameter of a single fiber of the porous backing layer is about 100 µm, a film thickness of slightly larger than this value cannot only meet the requirements of bonding strength, but also render an optimal combination of bending stiffness and flexibility.

An important feature of porous backing is the inconsistency of tensile and compression moduli, which ensures high adaptability during contact and equal load sharing during separation. Herein, porosity is an important parameter affecting tensile and compressive moduli, which is defined as the number of fibers per unit volume. Hence, the higher porosity corresponds to smaller pore size (Figure S16, Supporting Information). The tensile and compressive elastic moduli of porous structures with different porosities are shown in Figure 5d,e. It can be seen that the characteristics of low compressive modulus and high tensile modulus have not changed. We have also measured the adhesion strength of structures, with different porosities, to uneven surfaces, as shown in Figure 5f. Herein, the discretization units are all 4 × 4 and the cut depth is 2 mm to maintain consistency. The experimental data reflect that the low‐porosity structure, with a high ratio of tensile modulus to compressive modulus, exhibits a high adhesion strength, which can be attributed to the optimal balance of contact adaptability and pull‐out interfacial strength. It is worth noting that, if the porosity is further reduced, the number of fibers per unit area is gradually decreased, weakening the bonding strength between the supporting layer and backing layers. Hence, an optimal range of porosity should be considered for practical applications.

### 2.6 Technological Perspective

**Figure 6:**
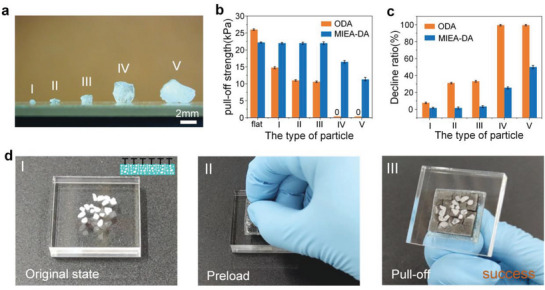
The adaptability of MIEA‐DA to mutant surfaces. a) The schematic diagram of particle size; b) the normal adhesion strength of both structures to different particles; c) the ratio of decline in adhesion strength with increasing particle diameter; and d) the grasping process of MIEA‐DA on the PMMA plate with particles.

In order to show the potential application field of MIEA‐DA, the particle surface was artificially set as the target object for performance testing, which is a typical form of mutant surface and easy to induce cracks during object manipulation. Mainly, SiO_2_ particles, with a diameter range of 500 µm to 4 mm, are distributed on the surface, as shown in **Figure** **6**a. These particles are placed on a glass slide and the normal adhesion strength of MIEA‐DA and ODA to it is tested by the load–pull process, as shown in Figure 6b. With the increase of particle diameter, the adhesion strength of ODA begins to decrease, indicating that the existence of particles accelerates the crack growth rate of the interface. In particular, when the particle diameter reaches >2 mm, the adhesion strength of ODA drops to zero, which means that it cannot achieve effective contact with the interface. In the case of MIEA‐DA, when the particle diameter reaches 4 mm, the adhesion strength is still 12 kPa, which is a significant improvement. The ratio of decline in adhesion strength with increasing particle diameter is shown in Figure 6c. It can be seen that the decrease rate of MIEA‐DA is much lower than ODA.

In order to show the grasping ability of MIEA‐DA on a particle surface, we have recorded the grasping process of MIEA‐DA on a PMMA plate with a lot of particles. As shown in Figure 6d, MIEA‐DA can still complete the grasping and particles are squeezed at the bottom of the discrete unit. The remaining discrete units maintain effective contact with the interface, which contributes to the adhesion (See Movie S9, Supporting Information). This stable operation ability on the particle surface greatly broadens the application range of dry adhesives, such as in some special dusty environments.

**Figure 7:**
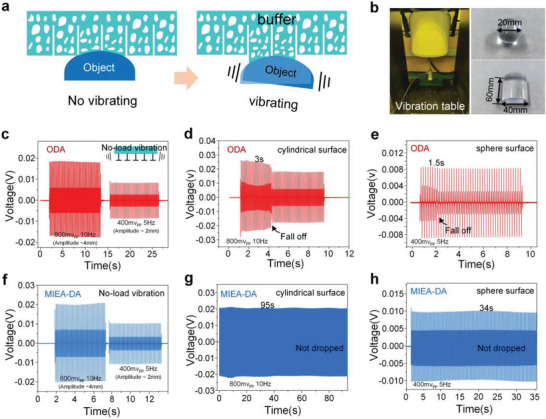
Verification of stable adhesion of multi‐stage discretized adhesion structure under different frequency vibrations. a) A schematic diagram of the anti‐vibrating characteristics of the adhesive structure during the process of surface adhesion; b) the schematic diagram of the test system and target objects; c–e) the anti‐vibration effect of ODA; and f–h) the anti‐vibration effect of MIEA‐DA.

In addition to effective adaptation to a mutant surface, coupling of cuts and porous features also endows MIEA‐DA adhesive with the ability to resist external vibrations. As shown in **Figure** **7**a, when an adhesive system is disturbed by vibrations, the pores can act as a buffer and partly absorb the vibrations‐induced energy. Moreover, owing to the existence of physical cuts, there will be no mechanical interactions, such as squeezing and friction, with each decoupling adhesion unit, ensuring the adhesion stability of the whole contact area. In order to verify the anti‐vibration performance of MIEA‐DA, a vibration platform is set to simulate the vibration environment, and typical cylindrical and spherical surfaces are selected as target objects, as shown in Figure 7b. During the test, the amplitude and frequency of the vibration platform are controlled by a signal generator (Figure S17, Supporting Information). First, ODA is fixed to the vibrator end of the vibration platform and the signal generator produces two square‐wave signals (800 mV_pp_/10 Hz and 400 mV_pp_/5 Hz), and the corresponding amplitudes are 2 and 4 mm, respectively. The waveform was recorded by an oscilloscope, as shown in Figure 7c. Both signals demonstrate uniform positive and negative fluctuations, and the amplitude of both signals is 0.02 and 0.01 mV, respectively. Then, the target object is placed under the adhesives to achieve stable adhesion and output vibrations are started. At this time, the waveform exhibits a falling edge after a few seconds, indicating the dropping of the target object. As shown in Figure 7d,e, the cylindrical and spherical surfaces are only held for 3 and 1.5 s, respectively. Figure 7f–h presents the anti‐vibration effect of MIEA‐DA, which is much better than ODA. For a cylindrical surface, there is still no obvious signal drop after being exposed to vibrations for 95 s, whereas the adhesion is maintained for 34 s for a spherical surface with a larger curvature (See Movie S10, Supporting Information). This significant improvement in anti‐vibration ability is conducive to the safe and stable applications of proposed adhesives in object manipulation.

## 3 Conclusion

In summary, a highly adaptive and stable dry adhesive is proposed based on multi‐scale bionics. Unlike the traditional bionic strategy based on terminal microstructure, the proposed strategy focuses on the synergistic influence of microscale contact end (seta), mesoscopic supporting layer (lamella), and macro backing (muscle tissues) of gecko's sole. In order to simulate the double discretization of seta on the soles of gecko on both micro‐ and mesoscales, mushroom‐shaped microstructure arrays are fabricated as contact ends, and physical cuts were introduced to form several independent adhesion units similar to the lamella. Also, a porous substrate is designed to achieve muscle‐like compliance. The coupling effect of interfacial mechanical decoupling realized by the cuts and as‐designed muscle‐like substrate endows the adhesive with high adaptability to complex irregular surfaces, even for that with abrupt contours. It is worth noting that the proposed method of cutting with a scalpel is a simple and scalable manufacturing method. In addition, since the scalpel is controlled with a mechanical device that can be positioned with micron accuracy, manufacturing repeatability can be guaranteed (Figure S18, Supporting Information). Of course, exploring new fabrication processes with more accurate cutting and controllability would better extend this innovative idea of interfacial mechanical decoupling.

The numerical simulations also confirm that the enhanced adhesion originates from the high compliance caused by the low elastic modulus incurred by porous feature and crack‐trapping effect due to the physical cuts. Besides, the inconsistent characteristics of tensile and compressive moduli caused by the uneven distribution of pores also play a positive role in the adhesion process. Moreover, the pores act as an ideal buffer to improve the anti‐vibration ability of the adhesive material by absorbing the damage shock from the external environment, expanding the application horizon of adhesive materials in grasping and transporting operations, which require high stability. The novel multi‐scale bionic strategy provides technical solutions for the engineering application of dry adhesives, and can further promote the artificial adhesive materials from the laboratory to real world.

## 4 Experimental Section

### Materials

Unless stated otherwise, solvents and chemicals were obtained commercially and used without further purification. The polydimethylsiloxane (PDMS) prepolymer and cross‐linker (Sylgard 184) were purchased from Dow Corning, USA. The Silicone Rubber (AB‐600) was purchased from Shenzhen Hongyejie Technology Co., Ltd., China. The polyurethane (PU) sponge was commercially purchased from Hangmei Company, China. The convex and concave surface was purchased from Changchun Jinlong Optics Co., Ltd., China.

### Fabrication of Mushroom‐Shaped Adhesive Structure

Mushroom‐shaped adhesive structure was made with PDMS through the currently commonly used molding process. In this paper, the mold was prepared by the double‐sided exposure process proposed by this team before, which can realize the fabrication of mushroom‐shaped structure with controllable structure and good uniformity. The body and the curing agent of the PDMS were mixed and stirred uniformly in a ratio of 10:1, and then poured onto the mold and evacuated for 10 min. Subsequently, spin coating was performed, and the curing was completed in an oven at 80 °C for 1 h. Finally, the structure was obtained by demolding. The thickness of backing layer was controlled by changing the casting parameters. The mushroom‐shape micro‐structures were about 14 µm height with a 12 µm spacing, and the diameter of contact tip and pillar were 18 and 14 µm, respectively.

### Fabrication of Different Kinds of Adhesives

The fabrication of the adhesives was mainly based on the molding process. First, a double‐sided exposure process was used to prepare a mold of adhesive structure, and then PDMS was poured on the surface of the mold, placed in a vacuum chamber for 10 min, and then spin‐coated at a speed of 2000 r min^−1^ for 40 s. Subsequently, a silicone rubber (AB‐600, *E* = 400 kPa) block with 2 cm × 2 cm × 4 mm was placed on the surface of the uncured PDMS, placed in a 90 °C oven for 1 h, and finally demolded to obtain ODA. A scalpel was used to cut the ODA surface to obtain DA. The silicone rubber block was replaced with a sponge block of the same size (2 cm × 2 cm × 4 mm), and the film was cut with a scalpel to obtain MIEA‐DA.

### Testing of the Normal Adhesion Performance

All normal adhesion tests used a tensile testing machine (Baoda, Shenzhen), and the loading and unloading speeds were both 5 mm min^−1^. The angular displacement platform can adjust the angle between the probe and the adhesive. CCD camera was used to observe the real‐time contact state between the adhesives and object surface.

### Testing of the Adhesion Performance to Surfaces with Height Error

The 100 µm thick glass was cut into 25 mm × 25 mm and 25 mm × 80 mm, and then the smaller glass was stacked on the larger glass surface to form a 100 µm high step surface. Different height errors (100–500 µm) can be obtained by adjusting the number of glass placed. During the test, the load–pull method was also adopted, and the loading and unloading speeds were both 5 mm min^−1^.

### Testing of the Adhesion Performance under Vibration

An electromechanical vibrator (SINOCERA JZK‐10) was used to apply a vibration with a certain frequency. An exciting signal from the function generator (Agilent 33220A) was amplified by a power amplifier (SINOCERA YE5871A) to drive the electromechanical vibrator. By controlling the waveform peak value and frequency of the signal generator, the intensity and frequency of the vibration signal can be changed. The oscilloscope was connected with the vibration head and can record the vibration signal in real time. The peak value of the vibration signal was closely related to the weight of the adhesion target, so the adhesion state of the interface can be described directly by signal analysis.

### Materials Characterization

The microstructure of the adhesive material was observed by SEM (SU8010, Hitachi, Japan). The adhesion ability of material was characterized by computer servo pull‐pressure test machine (PT‐1176, Baoda, China). The time‐varying contact state between the adhesive material and the probe was observed through a digital microscope (RS‐500C, Kone, China). The mechanical properties of the samples were tested by computer servo pull‐pressure test machine (PT‐1176, Baoda, China).

### Finite Element Simulation

The geometric model was a 2D form, which mainly included sphere probe and backing layer. The criterion of interface fracture damage was the maximum nominal stress, the specific parameters were 0.02, 0.02, and 0.02, the interface stiffness was 500 N mm^−1^, and the fracture energy was 1E‐5 mJ. In order to improve the convergence of the model, the viscosity coefficient of the interface was set to 1E‐12. In the finite element simulation, the same preload application can be achieved by adjusting the displacement of the loading stage to simulate the test situation in the experiment. The material property of the spherical probe was set to the linear elastic model: *E* = 55 GPa, *ν* = 0.25, and the material property of the backing layer was set to the Neo–Hookean model: *C*_10_ = 0.086, *D*_1_ = 0.972. For a systematic analysis of the simulation results, the reaction force, fracture energy, and contact area were set in the history output variables, and the normal contact stress and tangential contact stress were set in the field output variables. The stress distribution of the interface can be derived by setting the path on the contact interface.

## Conflict of Interest

The authors declare no conflict of interest.

## Author Contributions

J.S., H.T., and D.W. conceived the idea. H.T., D.W., and X.L. developed the materials and methods for the adhesive material. D.W. and J.Z. designed and performed the adhesion measurements. H.L. and X.C. designed and performed the mechanical performance testing. D.W., H.T., and X.L. analyzed the data. D.W., H.T., H.H., and C.W. performed the numerical simulation. J.S. supervised and directed the research. D.W. and H.T. wrote the manuscript.

## Supporting information

Supporting Information

Supplemental Movie 1

Supplemental Movie 2

Supplemental Movie 3

Supplemental Movie 4

Supplemental Movie 5

Supplemental Movie 6

Supplemental Movie 7

Supplemental Movie 8

Supplemental Movie 9

Supplemental Movie 10

## Data Availability

The data that support the findings of this study are available from the corresponding author upon reasonable request.
