# Supplementary material for: Bioinspired Dry Adhesives for Highly Adaptable and Stable Manipulating Irregular Objects under Vibration
Source: Adv Sci (Weinh). 2023 May 7;10(21):2302512. doi: 10.1002/advs.202302512 (PMC10375138; doi:10.1002/advs.202302512)
Supplement: Supplementary file 1 — Supporting Information [file ADVS-10-2302512-s005.pdf]

ADVANCED SCIENCE
Open Access

Supporting Information

for *Adv. Sci.*, DOI 10.1002/advs.202302512

Bioinspired Dry Adhesives for Highly Adaptable and Stable Manipulating Irregular Objects under Vibration

Duorui Wang, Hongmiao Tian, Haoran Liu, Jinyu Zhang, Hong Hu*, Xiangming Li, Chunhui Wang, Xiaoliang Chen and Jinyou Shao*

Supporting Information for

Bioinspired dry adhesives for highly adaptable and stable manipulating irregular objects under vibration

Duorui Wang[1,2], Hongmiao Tian[1], Haoran Liu[1], Jinyu Zhang[1], Hong Hu[1,3]*, Xiangming Li[1,2], Chunhui Wang[1], Xiaoliang Chen[1,2], and Jinyou Shao[1,2]*

D. Wang, H. Tian, H. Liu, J. Zhang, H. Hu, X. Li, C. Wang, X. Chen, J. Shao
Micro-and Nano-Technology Research Center
State Key Laboratory for Manufacturing Systems Engineering
Xi'an Jiaotong University
Xi'an, Shaanxi 710049, China.
Email: hmtian@xjtu.edu.cn; huhong_xjtu@163.com

D. Wang, X. Li, X. Chen, J. Shao
Frontier Institute of Science and Technology (FIST)
Xi'an Jiaotong University
Xi'an, Shaanxi 710049, China.

H. Hu
Institute of Textiles and Clothing

The Hong Kong Polytechnic University

Hong Kong SAR, China.

Email: honghu@polyu.edu.hk

Supplementary Text

Numerical Analysis

1. Geometric modeling:

To simulate dry adhesion, a cohesive zone model based on cohesive surface is used. In the down pressing phase, we use general contact to simulate contact, and in the pull-up phase, we introduce cohesive contact Property. The formula of the cohesive constitutive law is as follows:

$$\mathrm{F} = \begin{Bmatrix} F_n \\ F_S \\ F_t \end{Bmatrix} = A \begin{bmatrix} K_{nn} & K_{ns} & K_{nt} \\ K_{ns} & K_{ss} & K_{st} \\ K_{nt} & K_{st} & K_{tt} \end{bmatrix} \begin{Bmatrix} \delta_n \\ \delta_s \\ \delta_t \end{Bmatrix} = AK\delta \qquad (1)$$

Among them, F_n represents the normal separation force on the interface during the separation process, F_s and F_t are two shear separation forces. δ_n、δs、δ_t represent the opening displacement of the cohesive interface in three directions, *A* represents the total area of the interface; *K* represents the interface stiffness matrix. The initial damage criterion of the interface adopts the maximum nominal stress damage criterion, and its constitutive formula is:

$$\mathrm{Max}\left\{\frac{\sigma_n}{\sigma_{Nmax}}, \frac{\delta_s}{\delta_{smax}}, \frac{\delta_t}{\delta_{tmax}}\right\} = 1 \qquad (2)$$

Considering the biomimetic adhesive materials inspired by reptiles are often based on the action mechanism of van der Waals forces, a zero-thickness cohesive surface was used for finite element simulation in this paper. The geometric model was a two-dimensional form as follows, which mainly includes sphere probe and backing layer. The *R* of spherical probe was 15 mm, and the center of sphere aligned with center of backing layer. The backing layer was a simplified rectangular with a height (h_1) of 3 mm and a length (l_1) of 20 mm. For the discretized backing, the length and width of the substrate and the size of the spherical probe remain unchanged. The height (h_2) of a single discrete element is 2 mm, the width (l_2) is 1 mm, and the center-to-center distance (l_3) is 1.2 mm. For the coupled porous morphology and discrete backing, the pores are uniformly arranged, the pore diameter (*d*) is 0.18 mm, the center distance of the pores (a=b) is 0.25 mm.

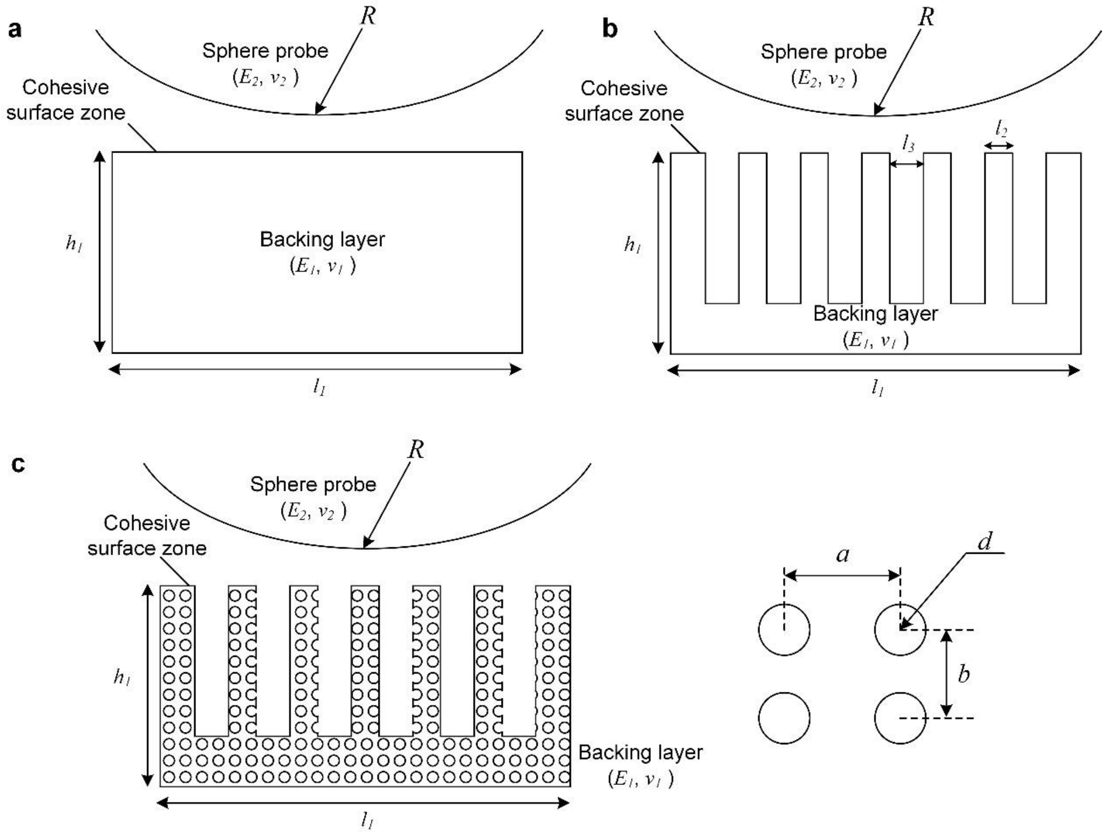

2. Boundary condition and interaction:

When exploring the numerical simulation of this separation process, the bottom of the substrate is fully immobilized. In the actual test, the test object first moved downward at a constant speed to contact the adhesive layer, and then moved upward at a constant speed until it was completely separated, so a constant displacement speed along the Y direction was applied on the upper surface of the spherical probe. In order to obtain the separation force-displacement curve more conveniently, a reference point is set at the center of the upper surface of the probe when the simulation model is established, and the upper surface of the probe is coupled with the reference point. At this time, applying a constant pulling speed at this point is equivalent to applying it to the entire upper surface.

In the ABAQUS/Standard contact analysis, the contact pair is often composed of the master surface and the slave surface, and the contact direction is always the normal direction of the master surface. Since the stiffness of the elastic backing is relatively small relative to the test probe, surface of the spherical probe was selected as the main surface, and the upper surface of the backing layer was the secondary surface. At the same time, due to the relative sliding or rotation between the two contact surfaces is small, small-slip contact is selected when defining the contact. The criterion of interface fracture damage is the maximum nominal stress, the specific parameters are 0.02, 0.02, 0.02, the interface stiffness is 500

N/mm, and the fracture energy is 1E-5 mJ. In order to improve the convergence of the model, the viscosity coefficient of the interface is set to 1E-12, the triangle diagram for cohesive mode is as follows:

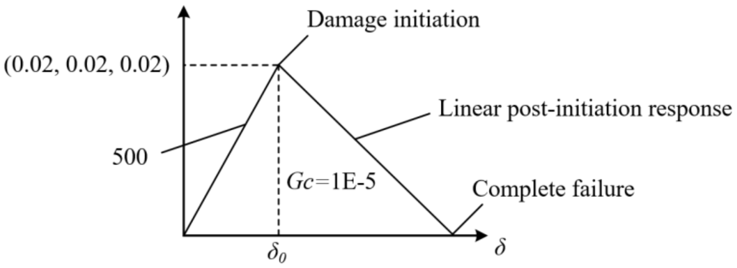

3. Simulation of adhesive behavior with different backing

In the finite element simulation, the same preload application can be achieved by adjusting the displacement of the loading stage to simulate the test situation in the experiment. The material property of the spherical probe was set to the linear elastic model: E=55 GPa, ν=0.25, and the material property of the backing layer is set to the Neo-Hookean model: C_{10}=0.086, D_1=0.972. For a systematic analysis of the simulation results, the reaction force, fracture energy, and contact area are set in the history output variables, and the normal contact stress and tangential contact stress are set in the field output variables. The stress distribution of the interface can be derived by setting the path on the contact interface.

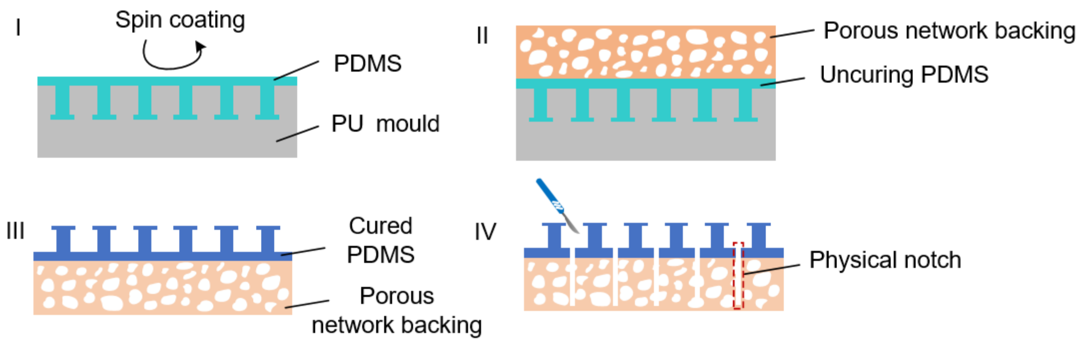

Figure S1. Detailed fabrication process of MIEA-DA. The porous backing adopts polyurethane foam with dimensions of 2 cm × 2 cm × 4 mm and an equivalent elastic modulus of approximately 80 kPa. The pore size inside the porous backing is about 500 μm and the diameter of the fiber skeleton inside porous structure is about 100 μm. In this paper, the PU mold with mushroom-like cavity was prepared by the double-sided exposure process proposed by our team before[44], which can realize the fabrication of mushroom-shaped structure with controllable topography and good uniformity. I: The pre-polymer and the curing agent of PDMS were mixed at 10:1 and then spin-coated on the surface of the PU mold at the speed of 2000 r/min. Then it was vacuum treated with 10 min, so that the PDMS is completely filled into the cavity of the mold. II: Attach the porous material to the mold surface at a certain pressure. III: The bonded porous substrate and mold were put into the oven and heated at 80 °C for 2 h to make the PDMS cured completely. IV: The contact end with mushroom-shaped structure can be obtained by demolding the porous material. Then the substrate was cut with a scalpel to realize the discretization. Each unit has a dimension of 5 mm × 5 mm and a depth of 4 mm, i.e. completely cut to the bottom.

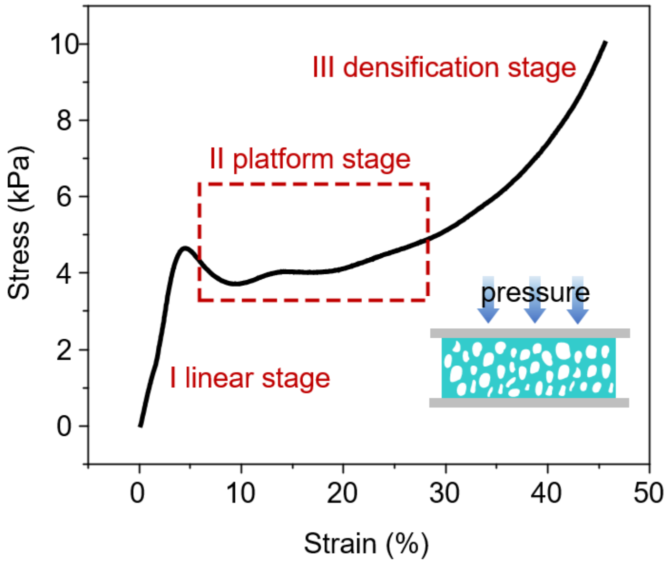

Figure S2. The strain-stress curve of the porous material under compression state. The area of the test sample is 2 cm × 2 cm and the pressing speed is 5 mm/min. With the increase of strain, the porous structure will go through three stages in turn. When the strain is 0-5%, the material is in the stage of linear deformation, and the stress increases linearly with the strain. When the strain is 5%-30%, the material will go through the platform stage, which is mainly manifested as the slowing down of the stress increase rate. When the strain is greater than 30%, the material enters the stage of densification deformation, and the stress increases rapidly with the strain.

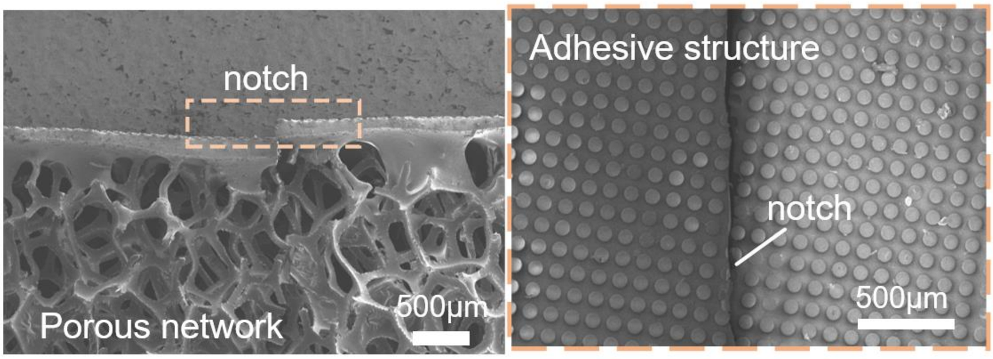

Figure S3. **The scanning electron microscope (SEM) image of MIEA-DA.** It can be clearly seen from the scanning electron microscope (SEM) images that MIEA-DA is composed of three parts, i.e., the mushroom-shaped microstructure on the top layer, a thin adhesive film separated by cuts, and a porous backing layer. The pore size inside the porous backing is about 500 μm and the diameter of a single fiber is about 100 μm.

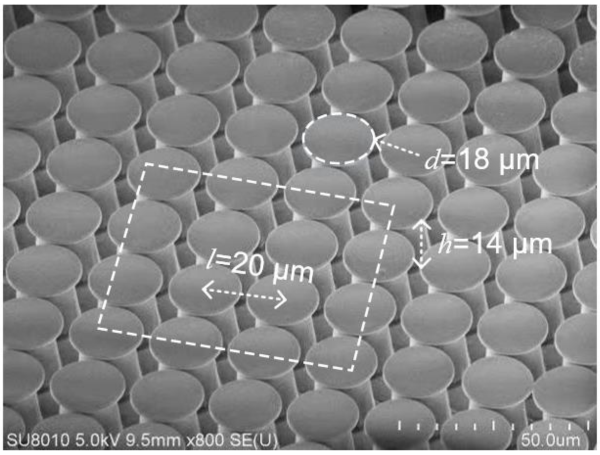

Figure S4. **The scanning electron microscope (SEM) image of mushroom shaped structure**. The mushroom-shaped tip was distributed in a square shape, and the diameter, height and spacing were 18 μm, 14 μm and 20 μm, respectively.

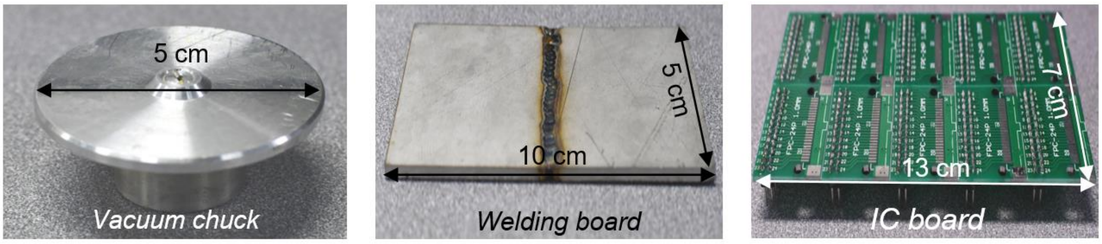

Figure S5. Dimensional characterization of several typical parts with abrupt contours. The abrupt features correspond to the pores on the surface of the vacuum chuck, the welding seam on the surface of the metal plate and the welding point on the surface of the IC board.

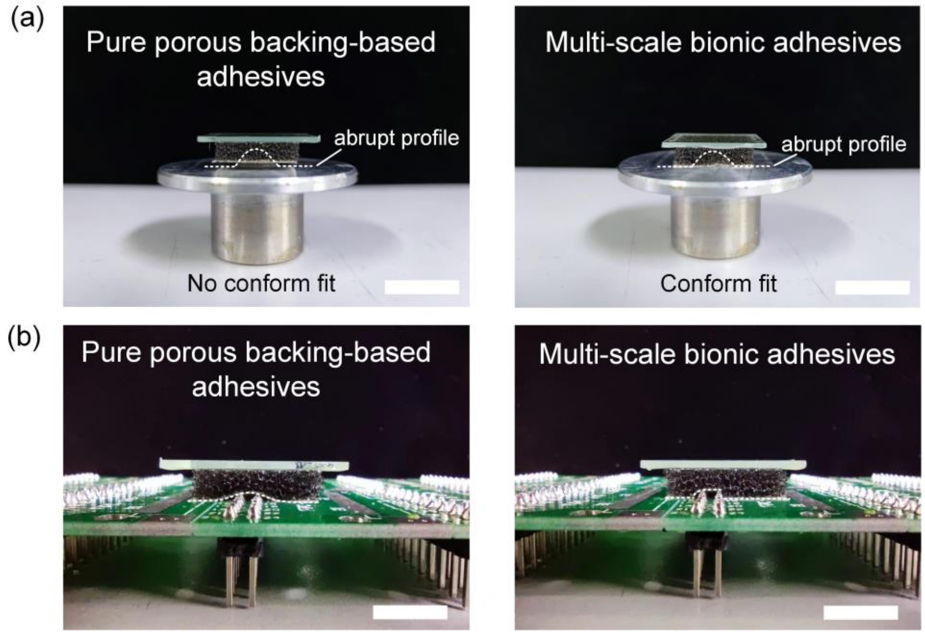

Figure S6. Description of the contact state of the adhesives with the target surface (a) vacuum chuck (scale bar = 2 cm) and (b) IC circuit board (scale bar = 1 cm). To characterize the contact state of different adhesives on abrupt contours, preload was applied to make contact between the adhesives and the vacuum chuck and the IC circuit board, and then the preload was removed and the immediate contact state was recorded at that moment. The results show that for vacuum chuck with more uneven contours, pure porous backing-based adhesives are unable to achieve a conform fit when the preload is removed due to the release of elastic energy; in contrast, multi-scale bionic adhesives can always achieve a conform fit with the target surface due to the mechanical decoupling of the interface, which helps to improve the adhesion performance. For IC circuit boards, although pure porous backing-based adhesives establish a certain contact area, the non-contact area caused by abrupt solder joints is significantly larger than that of multiscale bionic adhesives, which will inevitably cause a more rapid peeling effect during pulling-up, resulting in a weak adhesion effect.

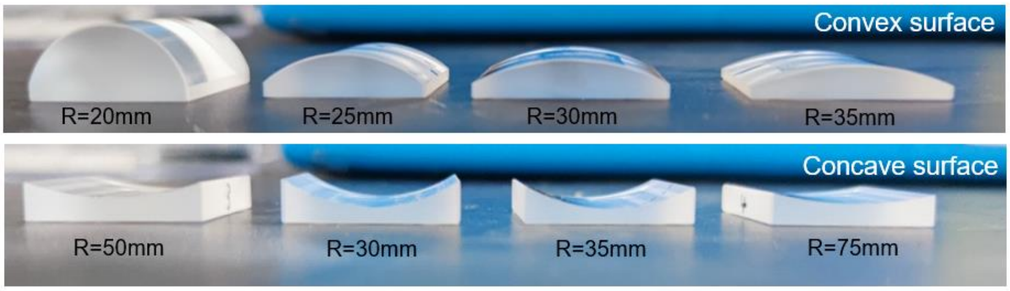

Figure S7. The size of the typical convex and concave surface. The material of the object is glass, and the length of the bottom surface is 30 mm. During the test, the convex or concave surface was placed on the loading table, and the adhesive sample was fixed with the probe of pull-pressure equipment.

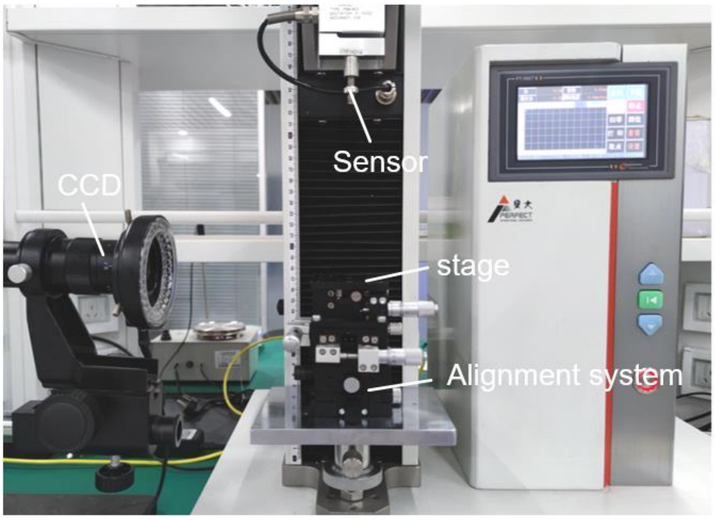

Figure S8. The physical map of the adhesion performance testing equipment. The testing system includes a mechanical sensor, a load stage, an angular displacement platform for alignment and a CCD. During the test process, the adhesive sample was fixed on the stage, and the object target was connected with the mechanical sensor. The probe was pressed against the sample with a defined preload and retracted in a standard load-displacement experiment. The pull-off force was defined as the maximum tensile force. The contact-separation state of the interface can be observed in real time through CCD.

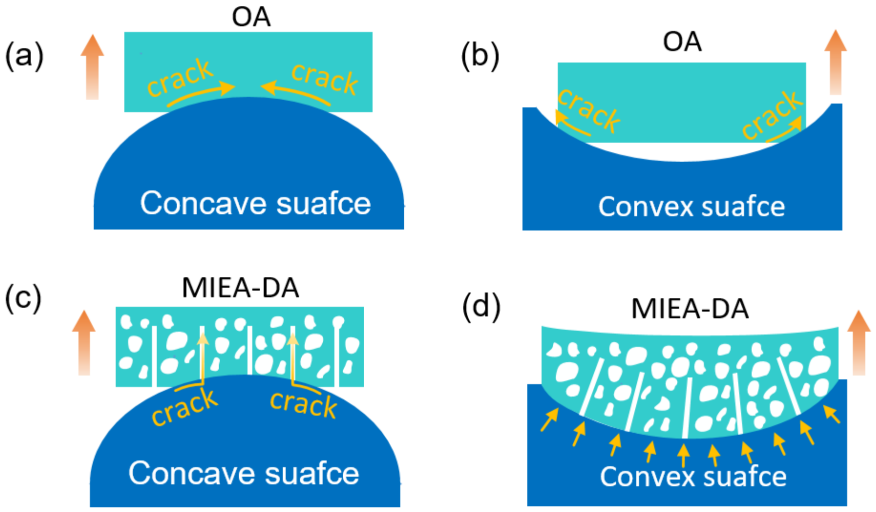

Figure S9. **The adhesion mode of the OA and MIEA-DA to different surface**. (a) The separation stage of OA to concave surface. At this moment, the crack will arise from both sides and propagate rapidly to the center for concave surface. (b) The separation stage of OA to convex surface. At this moment, due to the peeling effect caused by incomplete contact, the crack will arise from the center and propagate rapidly to both sides. (c) The separation stage of MIEA-DA to concave surface. At this moment, the crack will be restrained by the notch when it propagates to the central area, thus increasing the adhesion force significantly. (d) The separation stage of MIEA-DA to convex surface. Based on the conformal fit brought by the improvement of adaptability, the interface separation tends to be stretched as a whole, thus exhibiting a high adhesion strength.

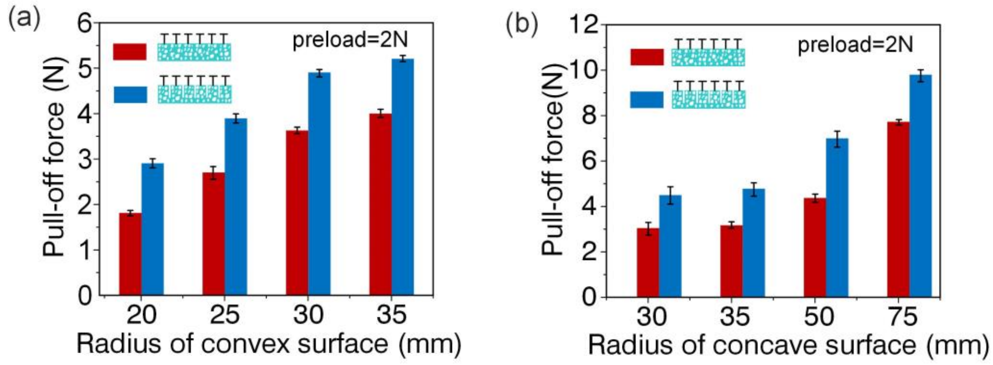

Figure S10. Comparison of adhesion performance of different adhesives on non-flat surfaces. (a) Adhesion performance of pure porous backing-based adhesives and MIEA-DA on convex surfaces with different curvatures; (b) Adhesion performance of pure porous backing-based adhesives and MIEA-DA on concave surfaces with different curvatures. The tests were performed in a typical load-pull manner and kept at the same preload (2 N). The results showed that the adhesive force of uncut porous backing to concave and convex surfaces with different curvatures are worse than that of MIEA-DA, which further demonstrates the importance of mechanical isolation.

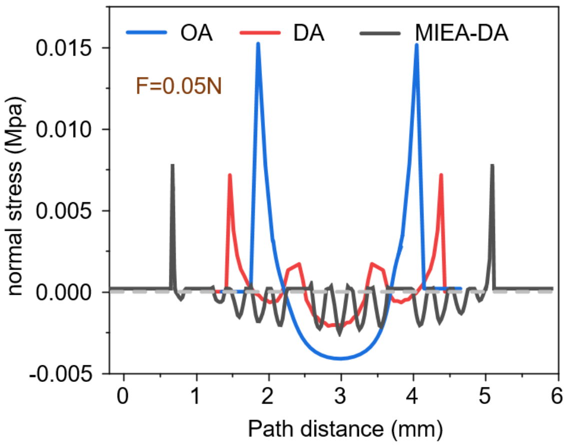

Figure S11. **The normal stress distribution at the interface of different adhesive structures under the same tensile load.** The results show that the three structures produce high stress concentration at both ends of the contact interface, and the contact area of MIEA-DA is the largest. In addition, due to the low tensile load (0.05 N), the compression deformation in the central area of the interface has not recovered, so it shows a compression state (negative value). Due to the porous characteristics, the interfacial stress distribution of MIEA-DA shows several peaks.

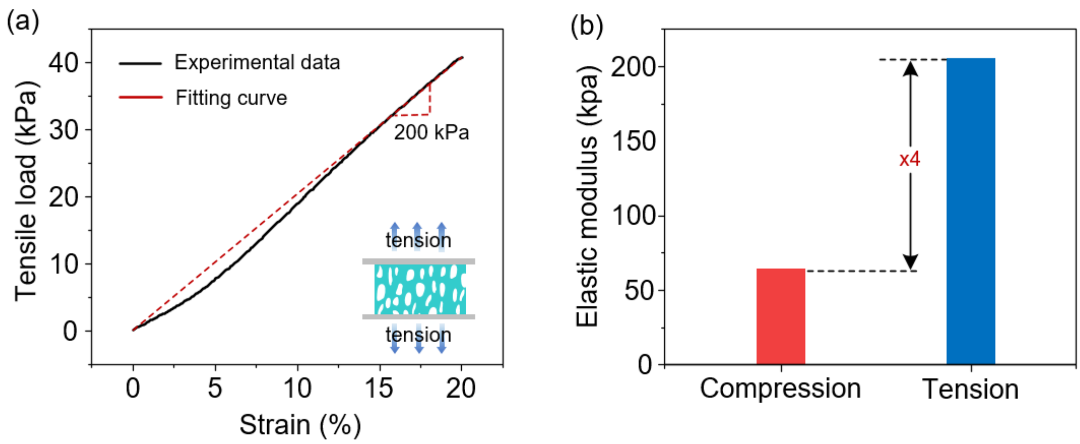

Figure S12. **The anisotropic mechanical properties of porous backing.** (a)The stress-strain curve of porous backing with 2 cm × 2 cm ×4 mm under tensile load. (b) Comparison of compressive modulus and tensile modulus of porous backing. It should be noted that the compression modulus is defined as the slope of the curve in the linear deformation stage.

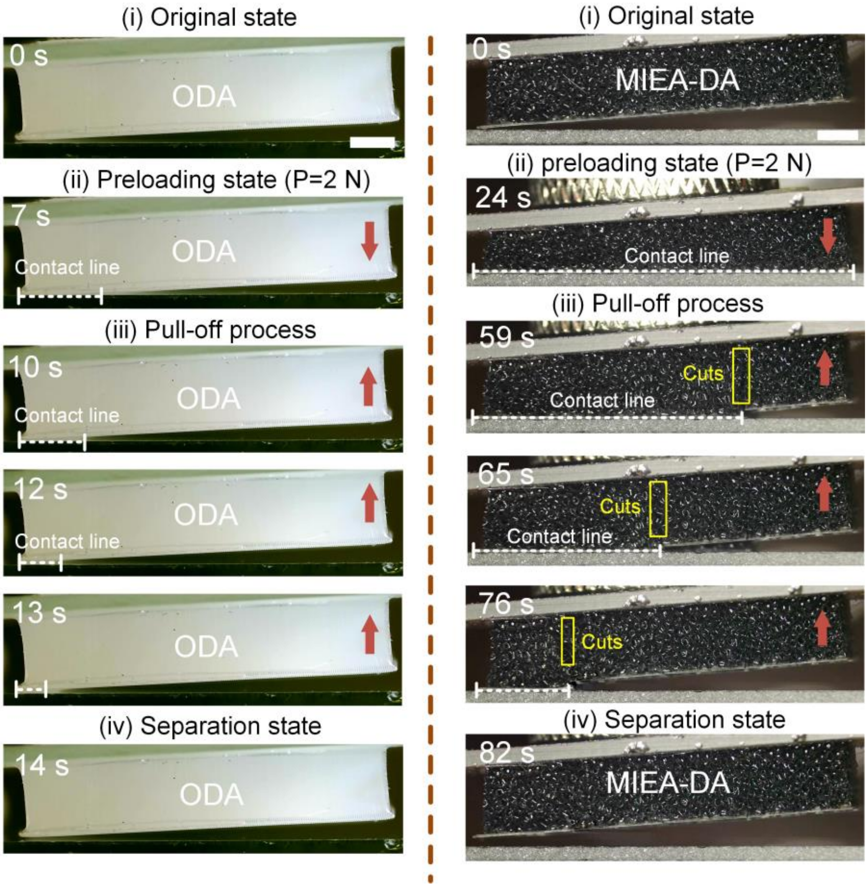

Figure S13. Time-series diagram of the contact separation process between the adhesives and the target surface in the case of an angle error of 3°. When the preload reached the set threshold (2 N), the contact area established between the ODA and the target surface was significantly lower than that of MIEA-DA, which established a complete ideal contact with the target surface due to a lower effective stiffness. Subsequently, during the pulling-up process, the continuous adhesion interface established by ODA in the previous do starts to peel off quickly and separates completely within a few seconds; in contrast, MIEA-DA shows a slow (up to tens of seconds) and discontinuous interfacial peeling process due to the mechanical isolation effect, and the crack propagation is effectively suppressed when it reach to the cuts, eventually exhibiting higher adhesion performance.

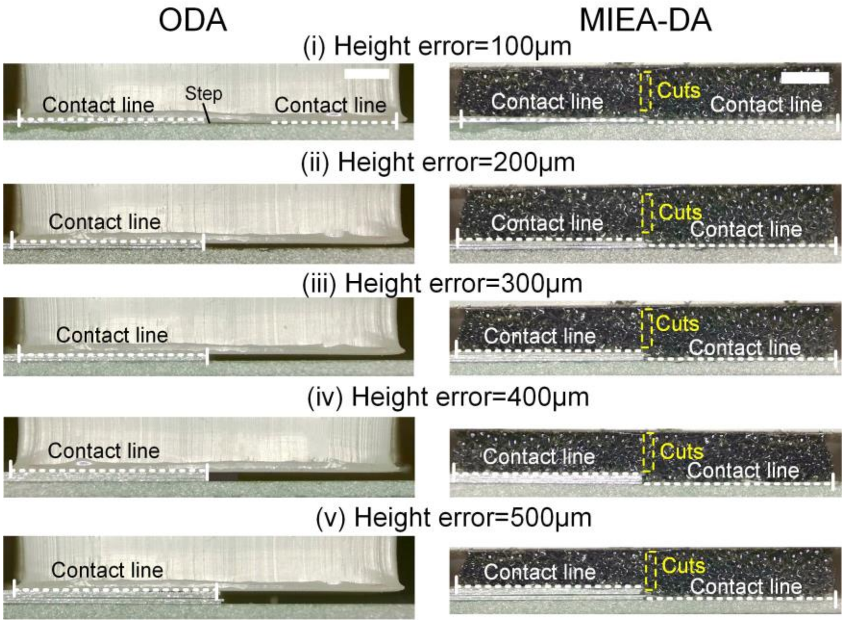

Figure S14. Screenshot of the contact area between different adhesives and the step surface under the same preload. When the step height is 100 μm, the ODA can establish most of the contact with the target surface, except for the step where there is a small loss of contact area due to elastic deformation. For MIEA-DA, the abrupt profile at the step can still be well adapted due to the presence of the cutout that decouples the entire interface mechanics. With the further increase of step height, ODA cannot establish an effective contact, while MIEA-DA can still maintain the ideal adaptive contact all the time by relying on its low modulus and the mechanical decoupling property of interface.

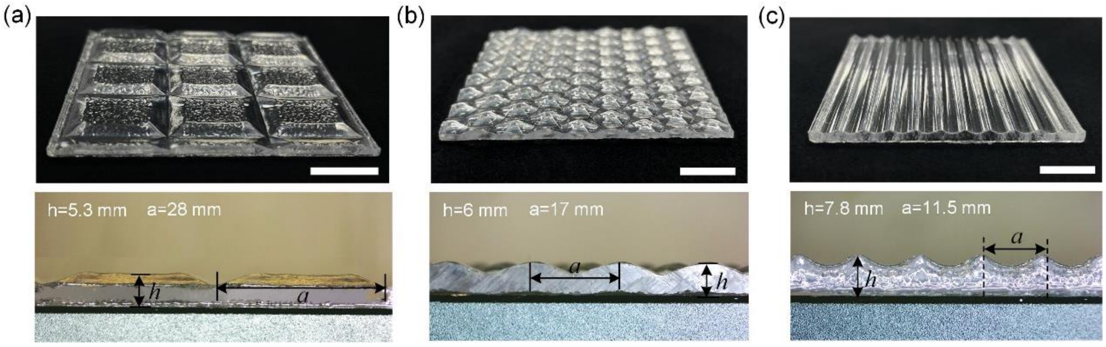

Figure S15. The Structural Characterization of Non-Flat surfaces with different feature arrays (scale bar = 1 mm).

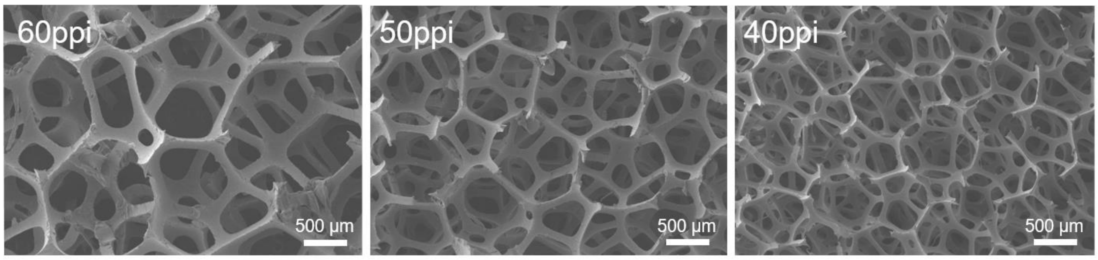

Figure S16. **Structural characterization of porous materials with different porosity.** Here, ppi is used to describe the porosity, which means the number of fibers per unit volume. The higher the ppi value, the less the number of fibers per unit volume and the looser the pore distribution.

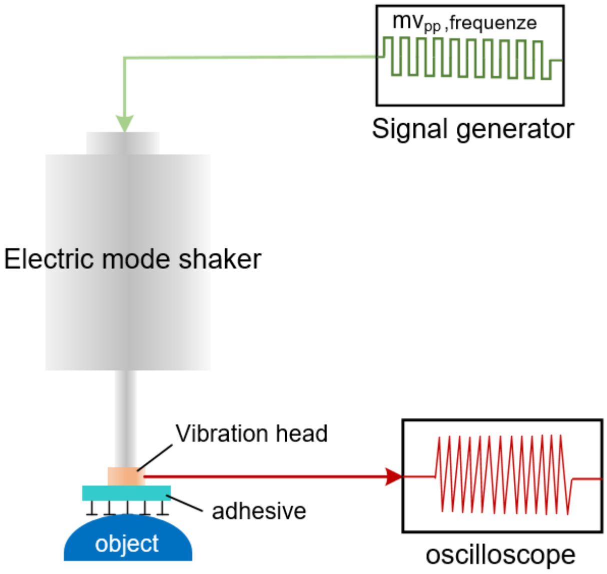

Figure S17. **The anisotropic mechanical properties of porous backing.** By controlling the waveform peak value and frequency of the signal generator, the intensity and frequency of the vibration signal can be changed. The oscilloscope is connected with the vibration head and can record the vibration signal in real time. The peak value of the vibration signal is closely related to the weight of the adhesion target, so the adhesion state of the interface can be described directly by signal analysis.

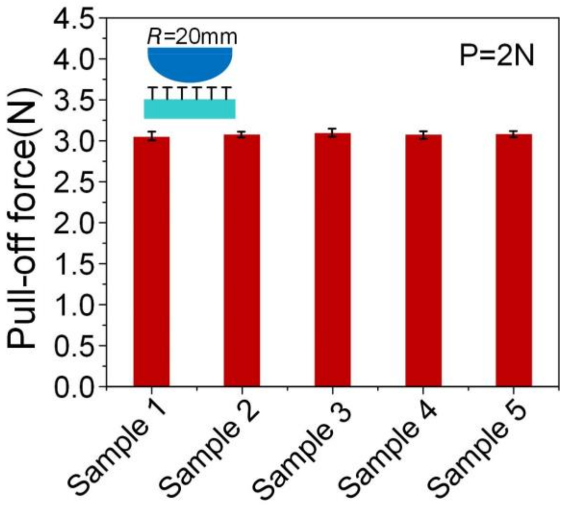

Figure S18. Effect of manufacturing process on experimental reproducibility. To investigate the effect of cutting with a scalpel on experimental reproducibility, five adhesive sample were fabricated in the same process and cut to the same discrete unit size. The adhesion properties were tested using a convex target with a radius of 20 mm, and the prelad was set to 2 N. The results showed that the adhesion variability between the different samples was not significant, with a maximum difference of no more than 10%.

Captions for Supplementary Videos

Supplementary Video 1 | Demonstration of several traditonal soft adhesives for grasping objects with abrupt contours.

Supplementary Video 2 | Demonstration of pure porous backing-based adhesives and MIEA-DA for grasping object with abrupt contours.

Supplementary Video 3 | Demonstration of the anti-vibration ability of plane adhesion (500g weight).

Supplementary Video 4 | Numerically dynamic contacting-separating behavior of the ordinary dry adhesives (ODA).

Supplementary Video 5 | Numerically dynamic contacting-separating behavior of the mechanically isolated dry adhesives (MIDA).

Supplementary Video 6 | Numerically dynamic contacting-separating behavior of the mechanically isolated energy-absorbing dry adhesives (MIEA-DA).

Supplementary Video 7 | The contact separation process between the adhesives and the target surface in the case of an angle error of 3 °.

Supplementary Video 8 | The contact separation process between the adhesives and the target surface in the case of a step height error of 200 μm

Supplementary Video 9 | Demonstration of the adaptability to surface with particles.

Supplementary Video 10 | Demonstration of the anti-vibration ability of curved surface adhesion.
